# Assessment of Gait Therapy Effectiveness in Patients with Parkinson’s Disease on the Basis of Three-Dimensional Movement Analysis

**DOI:** 10.3389/fneur.2016.00102

**Published:** 2016-06-27

**Authors:** Elżbieta Mirek, Jadwiga Lubomira Kubica, Jadwiga Szymura, Szymon Pasiut, Monika Rudzińska, Wiesław Chwała

**Affiliations:** ^1^Department of Clinical Rehabilitation, University of Physical Education, Krakow, Poland; ^2^Department of Physical Education and Sport, University of Physical Education, Krakow, Poland; ^3^Department of Neurology, Medical University of Silesia, Katowice, Poland

**Keywords:** Vicon 3D system, PD, gait, physical exercise

## Abstract

**Objective:**

The aim of this study was to assess the effect of physical exercise on gait pattern disorders, based on three-dimensional gait analysis in the sagittal plane in a group of people with Parkinson’s disease (PD).

**Methods:**

Thirty-two subjects with PD (14 women and 18 men; age: 50–75 years) were qualified for the study, which ran for 3 weeks and included 18 therapeutic sessions. Thirty-five control subjects were included in the research (13 women and 19 men; age: 52–77 years). Gait analysis using the Vicon 3D system took place in the Biokinetics Laboratory. The research group was tested before and after treatment, and the control group was tested once.

**Results:**

Comparing the average peak angle changes and average standard time results (% gait cycle) corresponding with angles of movement in the lumbar spine, cervical spine, elbow joint, and shoulder joint, statistically significant changes were observed. The study results are indicative of differences in spatiotemporal parameters and angular changes in gait for both groups. After therapeutic treatment, we observed improvement in the angular range of changes in thorax tilting, but there were no difference between the most affected and less affected side. For the cervical spine, a significant reduction in flexion during dual support was observed. The angular range of changes in shoulder joint was significant only in less affected shoulder during the initial contact (F1), terminal stance (F4), and terminal stance (F8) phases of gait (*p* < 0.05). After therapeutic treatment, significant angle and setting changes in the most affected limb of the elbow joint occurred during the initial contact and terminal swing phases (F1, F8). In the terminal stance phase (F4), an increase in range of motion by about ±4° was observed (*p* < 0.05).

**Conclusion:**

Exercise therapy slightly increased the range of movement in the examined joints of PD’s patients. Results of pathological walking patterns occurring prior to treatment improved after treatment and moved closer to the physiological gait pattern.

## Introduction

Postural instability, bradykinesia, hypokinesia/akinesia, rigidity, and tremors are clinical elements in the diagnosis of Parkinson’s disease (PD) (1). Reduced walking velocity, stride length, cadence, and proportion of swing time to stance time are common gait abnormalities, but motor deficits resulting from significant dopaminergic striatal denervation are present in both the upper and lower limbs (2).

The majority of research to date has been focused on the functioning of the lower extremities, while the movements of the upper and lower extremities are coordinated, any disruptions in inter-limb coordination may affect stability and lead to gait disturbances. As reported, arm swing is an essential component of locomotion, which may influence human gait stability and remain reduced for even several years prior to diagnosis of PD (3–6).

Studies revealed that normal arm swing increases global gait stability and may help the recovery of gait stability following perturbation. Elftman (5) reported that arm swing decreases angular momentum around the vertical and vertical ground reaction moment causing a minimization of energy consumption. In this manner, arm swings generate a horizontal torque to the upper trunk, and through the shoulder girdle, they work as a gravitational pendulum. The result of the functional division of the body into the passenger and locomotor proposed by this author, is that tilting the body becomes an important driving force during gait. Some studies tried to answer the question whether arm swing is actively controlled (driven by muscle activity) or rather passive (7, 8). This idea was confirmed by Hogue (9); however, later studies suggested largely passive swinging of the arms (6, 9, 10). The assumption of a partly passive arm swing arose from the idea of evolution adjustment after a change from quadrupedal to bipedal locomotion.

It has been reported that significant physiological differences between the left and right arm swing amplitudes exist in healthy subjects. The asymmetry can be larger in an arm affected by neurological pathology. Asymmetrical presentation of motor dysfunctions in PD occurs as a result of the asymmetric process of nigrostriatal dopaminergic denervation. Symptoms are more visible on the most affected side and deteriorate with the ongoing disease (5, 11). No compensation of the lesser affected side was observed in comparison to other studies (12). Decreased inter-limb coordination was observed in early stages and in advanced stages of the disease (2, 8, 13). As movements of the upper and lower limbs influence each other, rehabilitation of gait focused on improving amplitude of arm movements may be beneficial.

Under these circumstances, the aim of this study was to analyze the arm, head, and lumbar movement in locomotion patterns, according to the presence of symptoms of patients with PD, on the basis of three-dimensional analysis and to compare the effect of a training program on selected gait parameters.

## Materials and Methods

### Study Participants

Patients with PD attending the Neurological Department were recruited. After giving their written consent, the eligible patients were clinically examined by an experienced neurologist. The Hoehn and Yahr scale was used to assess the disease stage. Thirty-two subjects with PD (14 women and 18 men; 66 ± 8.7; age: 50–75 years) and 35 control subjects (16 women and 19 men; 64 ± 7.8; age: 52–77 years) were included in the study. The inclusion criteria were: idiopathic PD diagnosed according to the UK PD Society Brain Bank criteria, I–III stage of the disease according to the Hoehn and Yahr scale [we used (MA) for most affected side of a body and (LA) for less affected side of the body], continuous pharmacological treatment with no change in doses for the last 3 months, 50–80 years of age, absence of additional neurological disorders or severe musculoskeletal system problems; no contraindications to physical training, capacity to provide written informed consent for participation in the study. Exclusion criteria were dementia, uncontrolled hypertension, change in doses of pharmacological treatment during the study, additional neurological disorders or severe musculoskeletal system problems, lack of informed consent, and gait disorders, which did not allow to walk 15 m. The characteristics in each group are presented in Table 1.

**Table 1:** **The characteristic of groups: the experimental and the control**.

Characteristic		Experimental group		Control group	

		*N* mean (SD)	% median (min–max)	*N* mean (SD)	% median (min–max)
Gender	Female	14	43.75	16	45.71
	Male	18	56.25	19	54.29
Age (years)		66.03 (8.66)	68.00 (50–80)	64.00 (7.80)	66.50 (52–70)
Duration of disease (years)		5.70 (2.66)	5.50 (1.5–12)	–	–
H&Y scale	1	1	2.00 (1–3)	–	–
	1.5	7			
	2	14			
	2.5	3			
	3	7			
Weaker body side	Left	18	56.25	–	–
	Right	14	43.75		

All subjects were treated with l-DOPA preparations (Madopar, Sinemet, Nakom) with doses between 50 and 1550 mg/day. None of the subjects had participated in a therapeutic program before. The study protocol was approved by the Jagiellonian University Ethics Committee in Krakow (Poland), and procedures were performed in accordance with Helsinki Declaration.

### Experimental Protocol

Subjects, who met the criteria, took part in the research project, including the Vicon three-dimensional system gait analysis and physical exercise therapeutic treatment.

Exercise therapy was a part of a standard procedure during hospitalization. The research project ran for 3 weeks and included 18 therapeutic sessions with a physiotherapist (6 times a week, for 60 min). Therapy was set individually every morning, during the “on-phase” of the subjects. Gait analysis using the Vicon 3D system took place in Biokinetics Laboratory. The research group was tested before and after treatment, and the control group was tested once.

### Gait Analysis: Vicon 250 System

The Vicon 250 system allowed to record and to analyze motion patterns in three-dimensional space. This system was coupled with five infrared CCTV cameras, which worked at 120 frames/s frequency. Plastic balls with a diameter of 25 mm, called passive markers, covered with reflective material, were affixed to the tested person to reflect the position of the bone and joint axis landmarks. The filmed, two-dimensional flat image of the markers motion was sent to the data station, where based on comparison between images from at least two cameras, it was processed to spatial image. In this manner, the Vicon 250 system identified the location of markers as points in space. The changes recorded at the data station were analyzed by the system and sent to a PC, on which, further processing of collected research material was done.

Before the tests, passive markers were stuck to the patient’s skin at characteristic anthropometric points and at a certain distance from the symmetry center of the joint (Table 2). Then, patients performed gait at a natural, stable speed on a track length of about 15 m, allowing to capture at least 4 cycles of gait. The data from 15 cycles of gait of the patients derived from the Vicon 250 system were subjected to statistical analysis. Average gait patterns of individual patients with PD used to obtain the averaged gait pattern for the whole group before and after therapeutic treatment.

**Table 2:** **Set of markers (one side of the body)**.

Symbol	Marker name	Marker position
RFHD	Right front head	Located approximately over the left temple
RBHD	Right back head	Placed on the back of the head, roughly in the horizontal plane of the front head markers
C7	7th cervical vertebrae	Spinous process of the 7th cervical vertebrae
CLAV	Clavicule	Jugular notch where the clavicles meet the sternum
TH10	10th thoracic vertebrae	Spinous process of the 10th thoracic vertebrae
STRN	Sternum	Xiphoid process of the sternum
RSHO	Right shoulder	Placed on the acromioclavicular joint
RUPA	Right upper arm	Placed on the upper arm between the elbow and shoulder markers
RELB	Right elbow	Placed on lateral epicondyle approximating the elbow joint axis
RFRA	Right forearm	Placed on the lower arm between the wrist and elbow markers
RWRA	Right wrist marker A	Left wrist bar thumb side
RWRB	Right wrist marker B	Right wrist bar pinkie side
RFIN	Right fingers	Placed on the dorsum of the hand just below the head of the second metacarpal
RASI	Right ASIS	Placed directly over the right anterior superior iliac spine
SACR	Sacral wand	Placed on the skin midway between the posterior superior iliac spine (PSIS)

### Therapeutic Treatment

The aim of therapeutic treatment was to teach proper movement patterns and to prevent the consolidation of already present pathological motor patterns. This was attempted using individually tailored stimulation techniques such as verbal and visual contact, resistance, traction, and approximation. The conducted exercises were selected individually according to the Vicon first assessment and designed to reduce muscle tension, restore normal ranges of motion in the lumbar spine, cervical spine, shoulder joint, and elbow joint, strengthen weak muscles, and consciously control posture. This was followed by gait and balance training, which included distance walking, performance of activities of daily living, such as turning, changing position, and overcoming obstacles. Attention was given to trunk rotation and arms swing while walking with cueing of cadence to improve stride length and walking speed. Training of posture and balance reactions was performed while sitting, standing, or walking. Complex movements were split into sequential components.

Strategies for dealing with gait freezing and shuffling gait were taught to the patients using a variety of external stimuli methods. Elements of education for the patient and his family were added to the therapeutic treatment to learn how to cope with family, professional, and social life situations.

### Statistical Analysis

Normal distribution of variables obtained in the study was assessed using the Wilcoxon test and equal variances were assessed using the Levene Median test. The significance of differences in mean values of the variables was tested with *t*-tests (one-sample location test and repeated measurements test). Data are presented as mean ± SD, *p* < 0.05 was used to establish statistical significance, and data were analyzed using STATISTICA 10 (StatSoft^®^) software.

## Results

Comparison of the angular movements of lumbar spine, cervical spine, shoulder joint, and elbow joint in the sagittal plane, during gait in patients with PD, before and after treatment.

**Figure 1:**
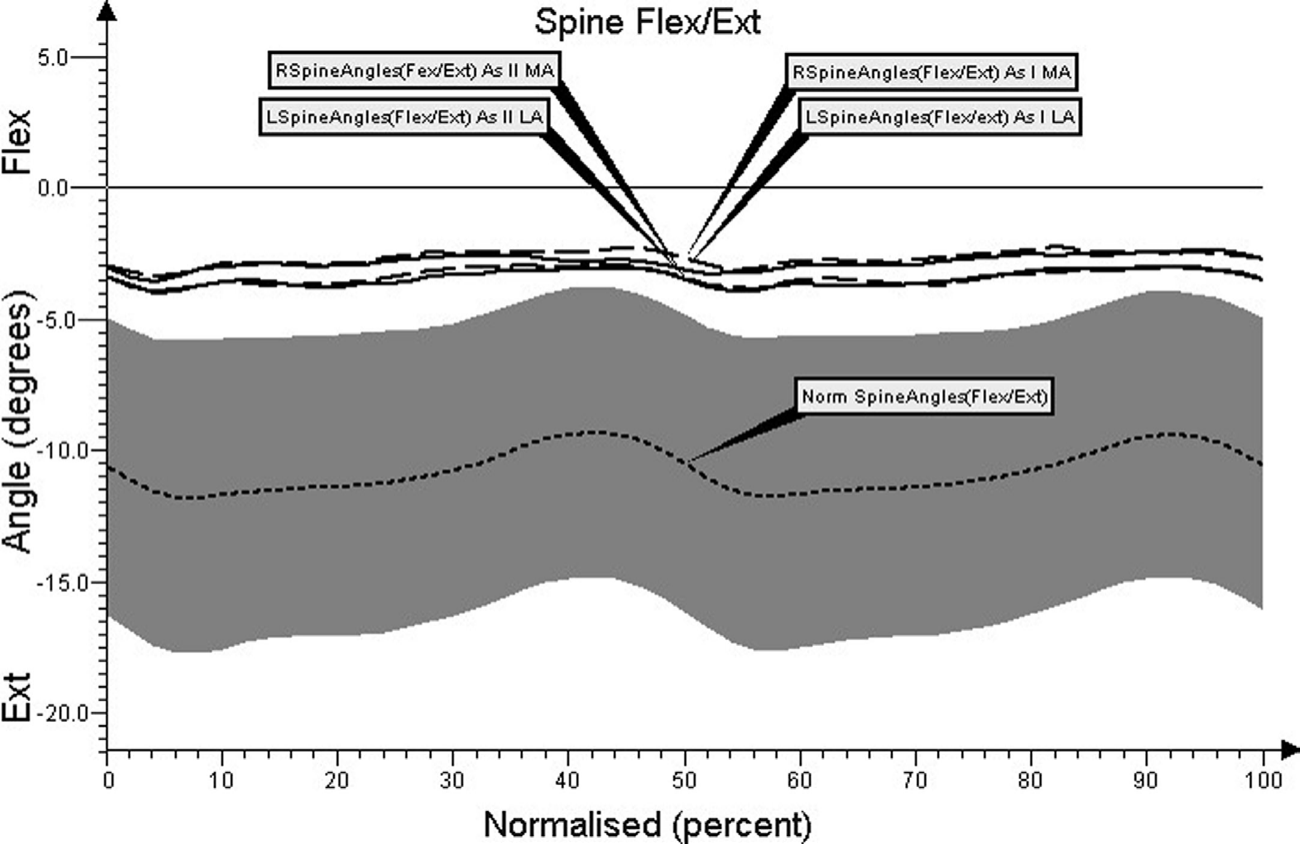
**Angular movement changes in the lumbar spine before and after treatment compared with control group**.

**Figure 2:**
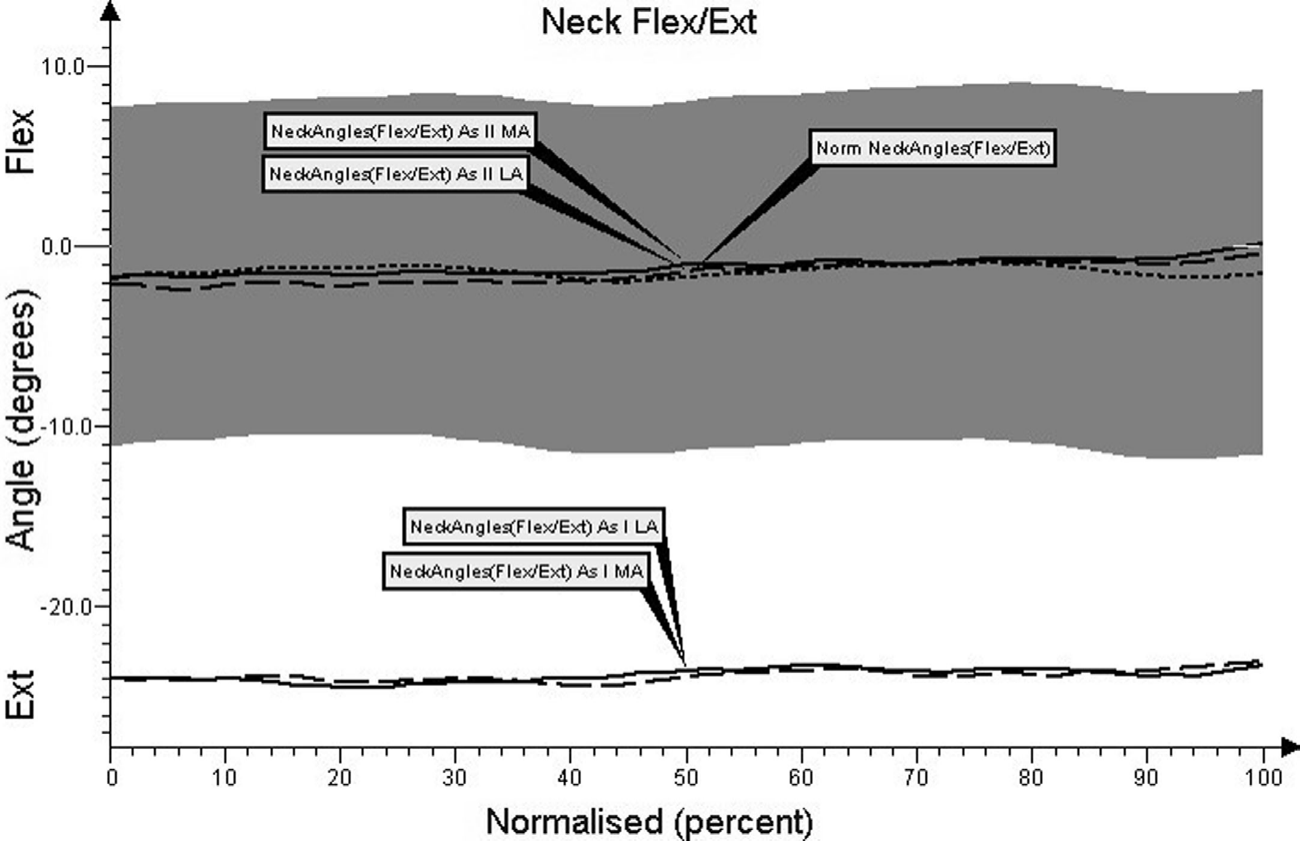
**Angular movement changes in the cervical spine before and after treatment compared with control group**.

**Figure 3:**
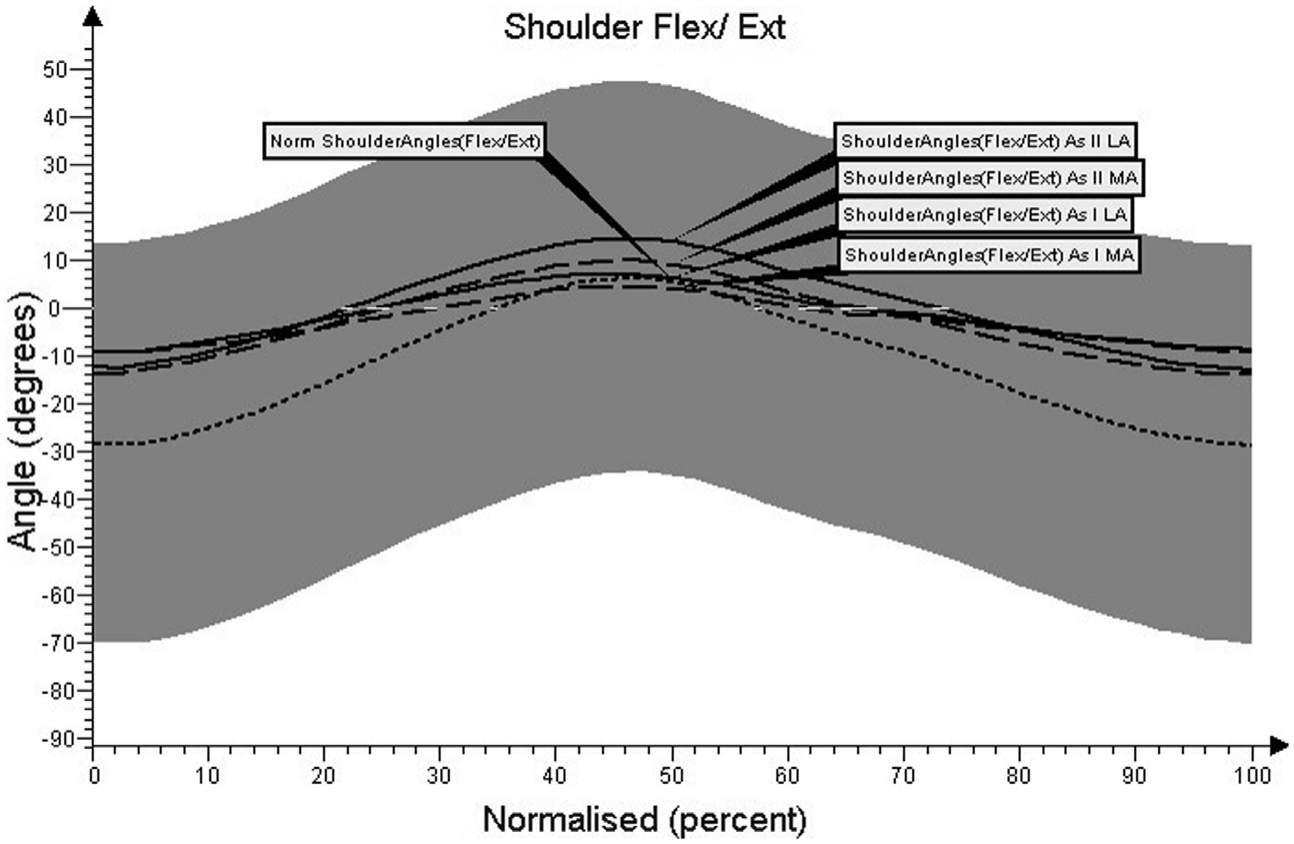
**Angular movement changes in the shoulder joint before and after treatment compared with control group**.

**Figure 4:**
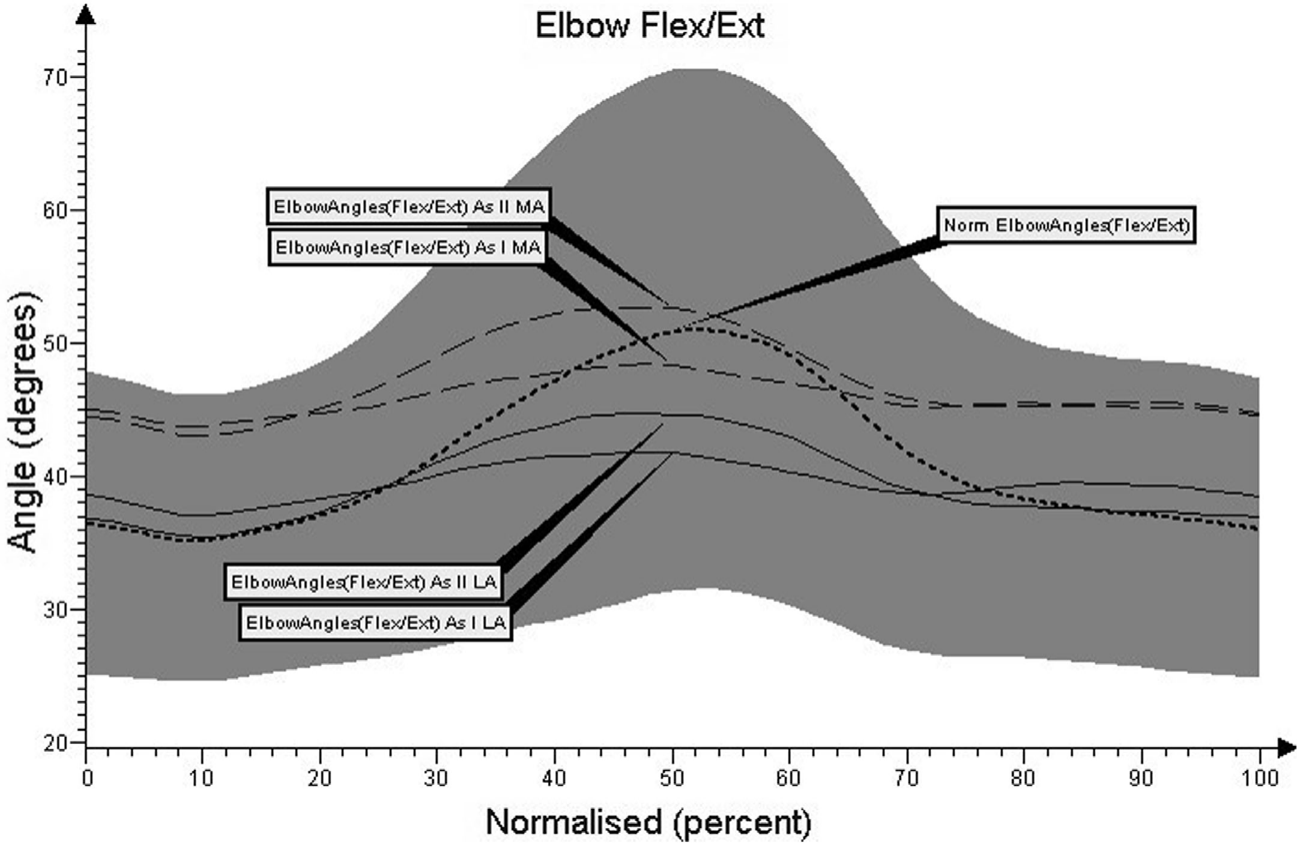
**Angular movement changes in the elbow joint before and after treatment compared with control group**.

Charts regarding the angular movement changes in the lumbar spine, cervical spine, shoulder joint, and elbow joint, before and after treatment in comparison to the control group are shown in Figures 1–4, respectively.

The study showed that during gait of PD patients, tilting of the thorax was clearly smaller than the mean value of control group’s tilting angle (difference of about 8°). Average values of chest tilting in the sagittal plane (Figure 1) did not differ significantly between the most affected side and the less affected side.

The course and setting angle of the curve changes in the cervical spine in patients with PD before treatment was flexion and differed from the control group (*p* < 0.05, Figure 2).

The significantly smaller range of angular changes in the most affected shoulder joint during the terminal stance (F4) phase of gait registered before therapy differed by about ±6°, compared with the healthy subjects (*p* < 0.05, Figure 3). Shoulder of the most affected upper limb had a tendency to maintain extended throughout the gait cycle.

The range of movement in the less affected elbow joint prior to therapy was similar to the range of double SD achieved by healthy subjects during every moment of the gait cycle (Figure 4). Angular changes among the analyzed curves did not differ significantly between groups for the elbow joint of the less affected limb and shoulder joint of the most affected limb in the first test (*p* < 0.05). Other curves for both the most affected and less affected limb differed significantly from the control group’s average (*p* < 0.001).

Analyzing the average angle of the thorax tilting before and after treatment, significant differences were found. During gait in PD patients, tilting of the thorax was clearly smaller than the mean value of control group’s tilting angle (*p* < 0.05). After therapeutic treatment, we observed improvement in the angular range of changes in thorax tilting. Average values of chest tilting in the sagittal plane (Figure 1) did not differ significantly between the most affected and less affected sides.

After therapeutic treatment, mean peak angular changes and standardized time referring to collected motion angles in the cervical spine were significantly different from the values before treatment (*p* < 0.05). Improvement in the results relates to the cervical spine setting and angular changes at different phases of the gait cycle (Figure 2). The curve for the test conducted after therapy concerning the most affected and less affected sides in the whole gait cycle, was close to the average achieved by a healthy person. The difference between the studies in terms of angle changes was about ±3°. A significant reduction in flexion during dual support was observed after therapeutic treatment.

The angular range of changes in the shoulder joint improved after therapeutic treatment, but the observed improvement was not statistically significant. The only significant changes were observed in the less affected shoulder during the initial contact (F1), terminal stance (F4), and terminal stance (F8) phases of gait (*p* < 0.05).

Significant angle and setting changes in the elbow joint occurred during initial contact and terminal swing phases (F1, F8) for the most affected limb before and after treatment (*p* < 0.05). In the terminal stance phase (F4), an increase in range of motion of about ±4° was observed after therapeutic treatment. The curve indicates that the nature of the movement was mainly flexion in all the phases of gait in patients with PD.

Angular changes among the analyzed curves did not differ significantly between groups for the shoulder joint of the most affected limb and the elbow joint of the less affected limb in the second test (*p* < 0.05). Other curves for both the most affected and less affected limb differed significantly from the control group’s average and between the first and second tests (*p* < 0.001).

## Discussion

Parkinson’s disease leads to motor dysfunctions in gait, speech, or writing. In recent years, particular attention was paid to gait abnormalities and the possibility of their improvement by pharmacological treatment and rehabilitation. The use of new, more accurate measuring devices, such as the Vicon system, allows to record and analyze motion in three-dimensional space (14, 15). Until now, there were few publications (16–18) related to the use of the Vicon motion analysis system in the assessment of therapeutic treatment effectiveness in patients with PD.

No studies were found on the upper extremities movement changes in connection to head and lumbar movements of patients with PD. Arm swing, which is an essential component of locomotion, may influence human gait stability (3–6). As the upper and lower extremities are coordinated, disruption in inter-limb coordination may lead to gait disturbances. Decreased inter-limb coordination can be observed at the early stages as well as advanced stages of the disease (2, 8, 13). Gait rehabilitation including improving the amplitude of arm movements may be beneficial.

Our study showed significantly reduced chest tilting during gait in patients with PD (Figure 1). This was probably associated with lower walking speed and resulted in reduction of body force driving developed from body ground reaction momentum. Such a situation may cause the need for greater muscle involvement in order to provide propulsion of the body. As studies revealed, decreased trunk mobility during walking may be present in individuals with PD (19). During locomotion, changes in coordination patterns have been observed for head and trunk motions. Flexible coupling of thoracolumbar and cervical segments allows locomotor task-dependent use of the upper limbs. This optimal coupling depends on walking speed, as arm swing compensates angular momentum with increasing gait velocity. The vertical moment to counter the torque from torso motion is produced by arm swing and is the only torsional effect (20, 21).

Our results show that the mean values of angular changes in the most affected shoulder joint did not differ significantly between patients and the control group. Results are within the range of double SD (Figure 3), but a greater range of extension was registered for the most affected shoulder joint at the terminal stance (F4) phase of gait. In the most affected elbow joint, statistically significant differences were registered during initial contact (F1), terminal stance (F4), and terminal swing (F8) in comparison to healthy subjects. This demonstrates the lack of balance on the most affected side. Similar parameters related to changes in the angle of the shoulder and elbow joints during gait were assessed by Knutsson (22) and Murray et al. (23), using the method of photokinemetric registration. The authors noted lack of coordination of the upper limbs, oversized shoulder extension, and greater elbow flexion in patients compared with the results obtained in the control group. In our study, following therapeutic treatment, an improvement in the range of shoulder and elbow flexion was observed. This points out the alternate work of upper limbs during gait, which did not exist prior to treatment.

There is no reference, in literature, to the results that we obtained in the present study, in which the impact of gait therapy treatment based on three-dimensional motion analysis on parkinsonian gait was evaluated, analyzing the angular changes corresponding to the recorded movements of the cervical spine. As follows from our analysis of the cervical spine movement curve during gait, limited range of extension prior to the treatment was affirmed. The conducted therapy had positive impact on changes in angular movements of the cervical spine, and the average course of the curve approached the course of the control group’s curve. There has been significant improvement in the obtained average values of reduction in peak flexion (extension) of cervical spine movement during the gait cycle. In control group, they occurred during the mid-stance (F3) phase of gait, and the average value of peak flexion occurred at the final (terminal) phase of gait. In patients with PD, these phases were delayed and occurred in terminal stance (F4) of cervical spine extension while flexion occurred during the initial swing (F6) phase.

Analysis of individual gait cycles of people with PD showed evident improvement in the angle of selected joint motion (24).

Studies by Wells et al. (25) revealed an increase in the range of motion of the upper limbs. In their study, the results of pathological walking patterns occurring prior to treatment improved after treatment and moved closer to the physiological gait pattern. Palmer et al. (26) claimed functional improvement in gait, coordination, and reduction of upper limb tremor after 3 months of therapeutic treatment; one focused on upper limb karate training and the other on trunk stretching exercises. Walking without arm swing is possible but requires greater effort of the legs due to greater foot moment reaction. As Park (27) referred, it is believed that arm swing in human walking minimizes torque loading on the joint and skeletal structure leading to optimization of lower limb motion. Whether motor learning affected by the neurodegenerative process in basal ganglia may be mediated by exercise was always a highlighted question, while studies have shown that rehabilitation positively affects structures “occupied” by disease (28, 29). According to the findings of Nieuwboer et al. (30) limits in motor learning caused by disease, extended rehabilitation, and learning processes are necessary to achieve automatization.

In studies on their own kinematic work patterns of the upper extremity joints, the lumbar and cervical spines are managed to be improved. In PD, the pathological gait pattern is fixed in the first years of the disease. Because of the already advanced changes in the musculoskeletal system, resulting from years of disease, it may be assumed that the duration of the therapeutic program should be longer to maintain favorable kinematic changes in the joint working pattern.

Assessing at exactly which point in time the gait phases occur, the corresponding values of movement ranges in the upper limbs joints, lumbar, and cervical spine in patients with PD rehabilitation program was elaborated taking into account all discussed abnormalities of gait pattern.

Patients with PD were characterized by reduced range of motion in the joints of the lumbar spine, cervical spine, shoulder, and elbow. Exercise therapy slightly increased the range of movement in the examined joints and also resulted in improved locomotion patterns. The results presented in the elaboration of their own research may be an example of very precise assessment of gait abnormalities, used for preparing gait rehabilitation and documenting the result progression after gait therapeutic treatment. It seems that the obtained results should contribute to further research penetration including a more representative group. Future studies should include a follow-up study to show whether the positive changes in locomotion last over a longer period of time.

### Limitation of This Study

We point out that a limitation of the current study was lack of patients assigned to a group with no treatment. Exercise therapy was a part of a standard procedure during hospitalization, and due to ethical considerations, group of patients with PD with no treatment have not been performed in the study.

## Author Contributions

EM: study design, data collection, data interpretation, manuscript preparation, and literature search. JK: data interpretation, manuscript preparation, and literature search. JS: statistical analysis, data interpretation, manuscript preparation, and literature search. SP: manuscript preparation and literature search. MR: data collection and medical supervision. WC: data collection and statistical analysis.

## Conflict of Interest Statement

The authors declare that the research was conducted in the absence of any commercial or financial relationships that could be construed as a potential conflict of interest.

## Abbreviations

angle, angular movement changes (degrees) in the sagittal plane; As I, assessment before treatment; As II, assessment after treatment; Ext, extension; Flex, flexion; LA, less affected side of a body; MA, most affected side of a body; Norm, angular variation observed during physiological gait in the control group; normalized, mean normalized time values (% cycle of gait).

## References

[1] Wolters ECH Laar T Berendse HW Parkinsonism and Related Disorders. Association of Parkinsonism and Related Disorders. Amsterdam: VU University Press (2010).

[2] Xuemei H Mahoney JM Lewis MM Du G Piazza SJ Cusumano JP Both coordination and symmetry of arm swing are reduced in Parkinson’s disease. Gait Posture (2012) 35:373–7. 10.1016/j.gaitpost.2011.10.180 22098825 PMC3297736

[3] Ortega JD Fehlman LA Farley CT Effects of aging and arm swing on metabolic cost of stability in human walking. J Biomech (2008) 41:3303–8. 10.1016/j.jbiomech.2008.06.039 18814873 PMC2741013

[4] Bruijn SM Meijer OG Beek PJ Dieen JH The effects of arm swing on human gait stability. J Exp Biol (2010) 213:3945–52. 10.1242/jeb.045112 21075935

[5] Elftman H The function of the arms in walking. Human Biol (1939) 11:529–35.

[6] Meyns P Bruijn SM Duysens J. The how and why of arm swing during human walking. Gait Posture (2013) 38:555–62. 10.1016/j.gaitpost.2013.02.006 23489950

[7] Rana AQ Saeed U Reduced arm swing 16 years before diagnosis of young onset Parkinson’s disease – a case report. Neurol Psychiatry Brain Res (2012) 18:120–1. 10.1016/j.npbr.2012.01.004

[8] Roggendorf J Chen S Baudrexel S Loo S Seifried C Hilker R Arm swing asymmetry in Parkinson’s disease measured with ultrasound based motion analysis during treadmill gait. Gait Posture (2012) 35:116–20. 10.1016/j.gaitpost.2011.08.020 21962405

[9] Hogue RE Upper-extremity muscular activity at different cadences and inclines during normal gait. Phys Ther (1969) 49:963–72. 5802700

[10] Kuhtz-Buschbeck JP Jing B. Activity of upper limb muscles during human walking. J Electromyogr Kinesiol (2012) 22:199–206. 10.1016/j.jelekin.2011.08.014 21945656

[11] Lewek MD Poole R Johnson J Halawa O Huang X Arm swing magnitude and asymmetry during gait in the early stages of Parkinson’s disease. Gait Posture (2010) 31:256–60. 10.1016/j.gaitpost.2009.10.013 19945285 PMC2818433

[12] Meyns P Van Gestel I Massaad F Desloovere K Molenaers G Duysens J. Arm swing during walking at different speeds in children with cerebral palsy and typically developing children. Res Dev Disabil (2011) 32:85–91. 10.1016/j.clinbiomech.2012.09.005 21531534

[13] Roemmich RT Field AM Elrod JM Stegemöler EL Okun MS Hass CJ Interlimb coordination is impaired during walking in persons with Parkinson’s disease. Clin Biomech (2013) 28:93–7. 10.1016/j.clinbiomech.2012.09.005 PMC3552037 23062816

[14] Carse B Meadows B Bowers R Rowe P Affordable clinical gait analysis: an assessment of the marker tracking accuracy of new low-cost optical 3D motion analysis system. Physiotherapy (2013) 99:347–51. 10.1016/j.physio.2013.03.001 23747027

[15] Barker S Craik R Freedman W Herrmann N Hilstrom H Accuracy, reliability and validity of spatiotemporal gait analysis system. Med Eng Phys (2006) 28:460–7. 10.1186/s12984-016-0115-z 16122966

[16] Picelli A Camin M Tinazzi M Vangelista A Cosentino A Fiaschi A Three-dimensional motion analysis of the effects of auditory cueing on gait pattern in patients with Parkinson’s disease: a preliminary investigation. Neurol Sci (2010) 31:423–30. 10.1007/s10072-010-0228-2 20182896

[17] Ayán C Cancela JM Gutiérrez-Santiago A Prieto I Effects of two different exercise programs on gait parameters in individuals with Parkinson’s disease: a pilot study. Gait Posture (2014) 39:648–51. 10.1016/j.gaitpost.2013.08 24021522

[18] Morris M Iansek R McGinley J Matyas T Huxham F Three-dimensional gait biomechanics in Parkinson’s disease: evidence for a centrally mediated amplitude regulation disorder. Mov Disord (2005) 20:40–50. 10.1002/mds.20278 15390033

[19] Adkin AL Bloem BR Allum JHJ Trunk sway measurements during stance and gait tasks in Parkinson’s disease. Gait Posture (2005) 22:240–9. 10.1016/j.gaitpost.2004.09.009 16278966

[20] Van Emmerik REA Wagenaar RC. Effects of walking velocity on relative phase dynamics in the trunk in human walking. J Biomech (1996) 9:1175–84. 10.1016/0021-9290(95)00128-X 8872274

[21] Dietz V. Quadrupedal coordination of bipedal gait: implications for movement disorders. J Neurol (2011) 258:1406–12. 10.1007/s00415-011-6063-4 21553270

[22] Knutsson E An analysis of parkinsonian gait. Brain (1972) 95:475–86. 10.1093/brain/95.3.475 4655275

[23] Murray MP Sepic SB Gardner GM Downs WJ. Walking patterns of men with parkinsonism. Am J Phys Med (1978) 57:278–94. 742658

[24] Mirek E Chwała W Longawa K Rudzińska M Adamkiewicz P Szczudlik A Proprioceptive neuromuscular facilitation method of therapeutic rehabilitation in the treatment of patients with Parkinson disease. Neurol Neurochir Pol (2003) 5:89–102. 15098336

[25] Wells MR Bosak A Scandalis T Werner W McCarty C Smutny C Gait alterations in patient with Parkinson’s disease in a physical treatment program. Gait Posture (1997) 6:120–33.

[26] Palmer SS Mortimer JA Webster DD Bistevins R Dickinson GL Exercise therapy for Parkinson’s disease. Arch Phys Med Rehabil (1986) 67:741–5. 10.1016/0003-9993(86)90007-9 3767624

[27] Park J. Synthesis of natural arm swing motion in human bipedal walking. J Biomech (2008) 41:1417–26. 10.1016/j.jbiomech.2008.02.031 18417138

[28] Keus SHJ Munneke M Nijkrake MJ Kwakkel G Bloem BR Physical therapy in Parkinson’s disease: evolution and future challenges. Mov Disord (2009) 24:1–14. 10.1002/mds.22141 18946880

[29] Tomlinson CL Patel S Meek CH Herd CP Clarke CE Stowe R Physiotherapy intervention in Parkinson’s disease: systematic review and meta-analysis. BMJ (2012) 345:e5004 10.1136/bmj.e5004 22867913 PMC3412755

[30] Nieuwboer A Rochester L Müncks L Swinnen SP Motor learning in Parkinson’s disease: limitations and potential for rehabilitation. Parkinsonism Relat Disord (2009) 1553:53–8. 10.1016/S1353-8020(09)70781-3 20083008

